# Heat shock protein 90 and calcineurin pathway inhibitors enhance the efficacy of triazoles against *Scedosporium prolificans* via induction of apoptosis

**DOI:** 10.15698/mic2014.06.150

**Published:** 2014-06-02

**Authors:** Fazal Shirazi, Dimitrios P. Kontoyiannis

**Affiliations:** 1 Department of Infectious Diseases, Infection Control and Employee Health, The University of Texas M.D. Anderson Cancer Center, Houston, TX 77030, U.S.A.

**Keywords:** apoptosis, 17AAG, calcineurin, itraconazole, posaconazole, reactive oxygen species

## Abstract

*Scedosporium prolificans* is a pathogenic mold resistant to current antifungals, and infection results in high mortality. Simultaneous targeting of both ergosterol biosynthesis and heat shock protein 90 (Hsp90) or the calcineurin pathway in *S. prolificans *may be an important strategy for enhancing the potency of antifungal agents. We hypothesized that the inactive triazoles posaconazole (PCZ) and itraconazole (ICZ) acquire fungicidal activity when combined with the calcineurin inhibitor tacrolimus (TCR) or Hsp90 inhibitor 17-demethoxy-17-(2-propenylamino) geldanamycin (17AAG). PCZ, ICZ, TCR and 17AAG alone were inactive *in vitro* against *S. prolificans* spores (MICs > 128 μg/ml). In contrast, MICs for PCZ or ICZ in combination with TCR or 17AAG (0.125-0.50 μg/ml) were much lower compared with drug alone. In addition PCZ and ICZ in combination with TCR or 17AAG became fungicidal. Because apoptosis is regulated by the calcineurin pathway in fungi and is under the control of Hsp90, we hypothesized that this synergistic fungicidal effect is mediated via apoptosis. This observed fungicidal activity was mediated by increased apoptosis of *S. prolificans* germlings, as evidenced by reactive oxygen species accumulation, decreased mitochondrial membrane potential, phosphatidylserine externalization, and DNA fragmentation. Furthermore, induction of caspase-like activity was correlated with TCR or 17AAG + PCZ/ICZ-induced cell death. In conclusion, we report for the first time that PCZ or ICZ in combination with TCR or 17AAG renders *S. prolificans* exquisitely sensitive to PCZ or ICZ via apoptosis. This finding may stimulate the development of new therapeutic strategies for patients infected with this recalcitrant fungus.

## INTRODUCTION

*Scedosporium prolificans* is an emerging filamentous fungus that causes severe, frequently fatal pulmonary or disseminated opportunistic infections in immunocompromised patients 1. *S. prolificans *is inherently resistant to treatment with a wide range of antifungals, including the new generation of broad-spectrum triazoles 1,2,3,4. Hence, new therapeutic strategies for *Scedosporium *infections are urgently needed.

In pathogenic fungi, the calcineurin pathway and heat shock protein 90 (Hsp90) play major roles in maintaining fungal homeostatic cell responses, including resistance to antifungal agents 5,6,7,8,9,10. The calcineurin inhibitor tacrolimus (TCR) is an immunosuppressive agent widely used in solid organ and hematopoietic stem cell transplant recipients to prevent graft rejection 11. TCR binds to the intracellular protein immunophilin FKB12 and forms a complex, thereby inhibiting activation of the calcineurin pathway. *In vitro* studies have suggested synergy between triazoles and calcineurin inhibitors against *Aspergillus* spp. and the Mucorales 12,13,14. Our group recently reported that treatment with the combination of TCR and posaconazole (PCZ) improves control of invasive, necrotizing cutaneous mucormycosis in immunosuppressed mice compared with PCZ alone 15.

Hsp90 is a molecular chaperone involved in stress responses of *Candida albicans* and *Aspergillus* spp. and plays a major role in echinocandin resistance via regulation of the calcineurin pathway 6,16. Specifically, pharmacological inhibition of Hsp90 by 17-demethoxy-17-(2-propenylamino) geldanamycin (17AAG) prevents azole resistance and abrogates this resistance in *C. albicans* and *A. fumigatus* in a human host 6,16. In addition, researchers recently suggested a role for the calcineurin pathway in regulation of apoptosis in fungi 17,18. However, the role of Hsp90 in apoptosis remains unclear. Therefore, simultaneous targeting of both ergosterol biosynthesis and Hsp90 or calcineurin pathways in *S. prolificans *may be an important strategy for restoring the potency of antifungal agents. Specifically, we hypothesized that TCR or 17AAG in combination with the triazoles PCZ or itraconazole (ICZ) induces apoptosis in *S. prolificans*. Thus, we examined the effects of TCR and 17AAG co-administration on PCZ and ICZ activity using several *in vitro* methods to evaluate induction of apoptosis in *S. prolificans.*

## RESULTS

### PCZ and ICZ are inactive when used alone against *S. prolificans*, but exhibit significant fungicidal activity when combined with TCR or 17AAG

**Figure 1:**
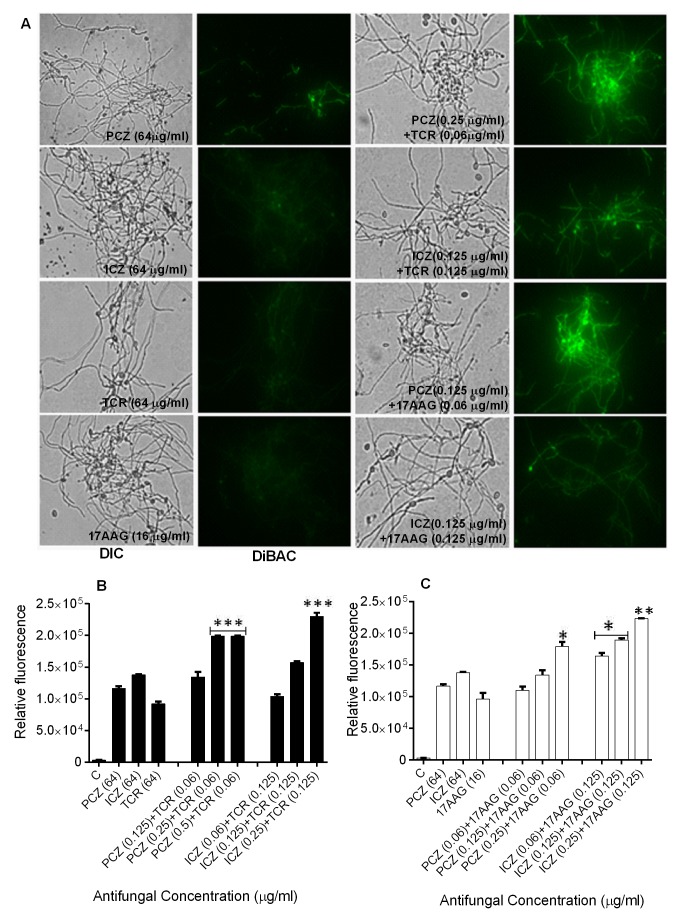
FIGURE 1: Fungicidal action of PCZ and ICZ alone and in combination with TCR and 17AAG against *S. prolificans *germlings (isolate 1). **(A)** Fluorescent images of *S. prolificans* germlings stained with the morbidity dye DiBAC. **(B, C) **Relative fluorescence levels in *S. prolificans* germlings treated with PCZ or ICZ plus TCR **(B)** or 17AAG **(C)** as determined using DiBAC staining. The experiments were performed in triplicate and repeated three times. *p<0.05; **p<0.001; ***p<0.0001 (compared with untreated control germlings and germlings exposed to drug alone). Error bars on graphs indicate standard deviation. DIC, differential interference contrast.

Individually, PCZ, ICZ, TCR, and 17AAG were inactive against *S. prolificans* (isolates 1 to 3)*,* with minimum inhibitory concentrations (MICs) ranging from 32 to 128 μg/ml. In contrast, the combination of PCZ or ICZ with either TCR or 17AAG rendered *S. prolificans* exquisitely more sensitive to the triazoles than did use of the triazoles alone (Table 1). Specifically, in combination with TCR or 17AAG, PCZ and ICZ were synergistic, with a fractional inhibitory concentration index (ΣFIC) of 0.5. In addition, bis-[1,3-dibutylbarbituric acid] trimethine oxonol (DiBAC) vital staining revealed enhanced uptake of stain and plasma membrane damage in *S. prolificans* germlings (isolates 1 and 2) exposed to PCZ or ICZ in combination with TCR or 17AAG (Figure 1 A-C; Table S1). Use of PCZ or ICZ (0.125-0.25 μg/ml) in combination with TCR or 17AAG resulted in 2.0- to 2.5-fold greater plasma membrane damage than did the use of triazoles alone.

**Table 1:** *In vitro *antimicrobial activity of PCZ and ICZ in combination with TCR or 17AAG against *S. prolificans* isolates. *MFC is given in parenthesis.

**Drugs**	**MIC (μg/ml)**		
	**Isolate 1**	**Isolate 2**	**Isolate 3**
PCZ	128 (>128)*	128 (>128)	128 (>128)
ICZ	128 (>128)	128 (>128)	128 (>128)
TCR	128 (>128)	128 (>128)	128 (>128)
17AAG	32 (128)	64 (>128)	64 (>128)
PCZ + TCR (0.06 μg/ml)	0.50 (0.50)	0.25 (1.00)	0.25 (0.50)
ICZ + TCR (0.125 μg/ml)	0.25 (1.00)	0.25 (0.50)	0.25 (0.50)
PCZ + 17AAG (0.06 μg/ml)	0.25 (0.50)	0.25 (1.00)	0.25 (0.50)
ICZ + 17AAG (0.125 μg/ml)	0.125 (0.50)	0.125 (0.50)	0.125 (0.50)

### Detection of intracellular Reactive Oxygen Species (ROS) accumulation and loss of mitochondrial membrane potential (ΔΨ_m_) in *S. prolificans *(isolates 1 and 2) germlings in response to treatment with PCZ or ICZ combined with TCR or 17AAG

**Figure 2:**
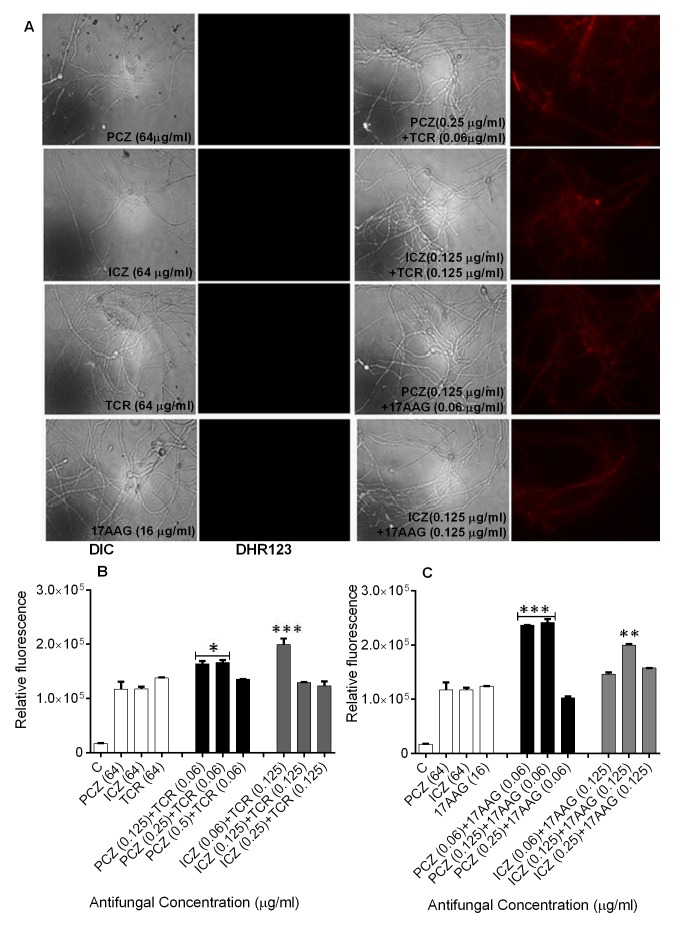
FIGURE 2: Intracellular ROS accumulation as detected by DHR-123 in *S. prolificans* isolate 1 germlings treated with PCZ or ICZ with either TCR or 17AAG, was measured using fluorescence spectrophotometry. **(A)** Fluorescent images of *S. prolificans* stained with DHR-123. **(B)** Relative fluorescence levels in *S. prolificans* germlings treated with PCZ or ICZ in combination with TCR. **(C) **Measurement of fluorescence of germlings treated with PCZ or ICZ in combination with 17AAG. *p<0.05; **p<0.001; ***p<0.0001. Error bars on graphs indicate standard deviation. DIC, differential interference contrast.

**Figure 3:**
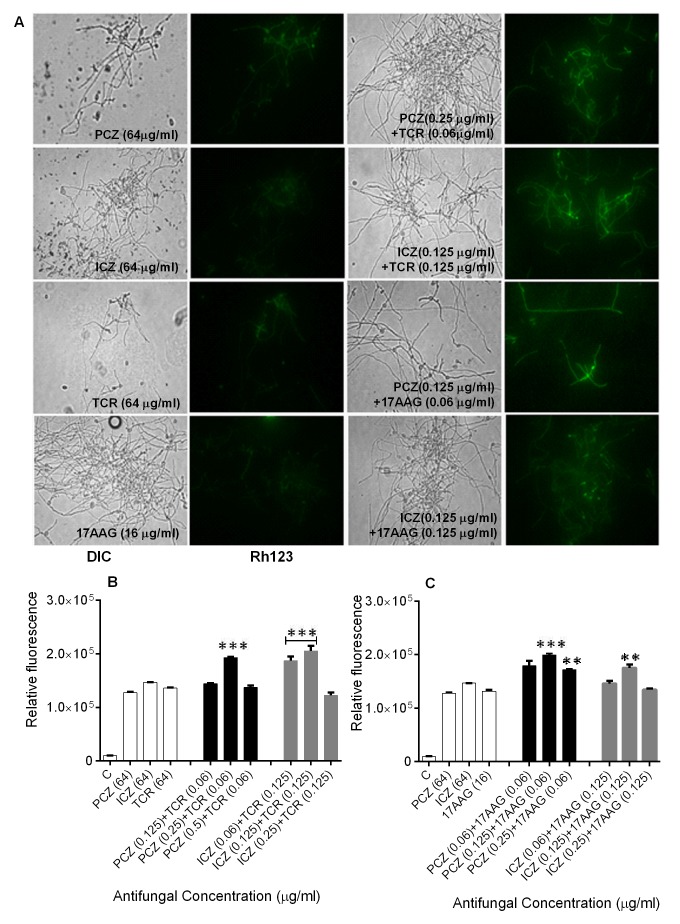
FIGURE 3: Changes in ΔΨ_m_ in *S. prolificans* isolate 1 germlings triggered by treatment with PCZ or ICZ combined with TCR or 17AAG. **(A)** Fluorescence images of *S. prolificans* germlings stained with Rh-123. **(B, C)** Relative fluorescence levels in *S. prolificans* germlings treated with PCZ or ICZ plus TCR **(B)** and PCZ or ICZ plus 17AAG **(C)**. **p<0.001; ***p<0.0001. Error bars on graphs indicate standard deviation. DIC, differential interference contrast.

Staining of *S. prolificans *germlings with dihydrorhodamine (DHR)-123 (red fluorescence) and rhodamine (Rh)-123 (green fluorescence) was most prominent in germlings treated with PCZ or ICZ in combination with TCR or 17AAG (Figures 2 and 3). A small percentage of control germlings and germlings treated with PCZ or ICZ alone exhibited positive staining for DHR-123 and Rh-123 (Figures 2 and 3). Staining with DHR123 and Rh-123 increased markedly when triazoles were combined with TCR or 17AAG, respectively (1.2-2.1 fold increase in fluorescence intensity), compared with triazoles alone (Figures 2 and 3 A-C). Isolate 2 in particular, had 1.0-2.1 fold and 1.3-2.1 fold increase in fluorescence for ROS accumulation and loss of mitochondrial potential, respectively, over germlings treated with triazoles alone (Table S1). Accumulation of intracellular ROS and disruption of ΔΨ_m_ are important steps in mitochondria-mediated apoptosis. These data indicate that treatment with PCZ or ICZ combined with TCR or 17AAG can trigger apoptosis in *S.* *prolificans *due to accumulation of ROS.

### Evidence of apoptosis in *S. prolificans* (isolates 1 and 2) induced by treatment with PCZ or ICZ in combination with TCR or 17AAG

Because various drugs can induce both apoptosis and necrosis in mammalian cells 19, we sought to differentiate between apoptotic and necrotic *S. prolificans *protoplast using annexin V-fluorescein isothiocyanate (FITC)-propidium iodide (PI) double staining, in which apoptotic cells are stained with annexin V-FITC (green), whereas the nuclei of necrotic cells are stained with PI (red) 20,21,22. Incubation of *S. prolificans *(isolate 1) protoplasts in the presence of PCZ (0.25 μg/ml) or ICZ (0.125 μg/ml) in combination with TCR (0.060-0.125 μg/ml) at 37°C for 3 h led to annexin V-FITC staining of 35-50% of the protoplasts. We found that 30-40% of protoplasts exhibited annexin V-FITC staining, when incubated with PCZ or ICZ in combination with 17AAG (0.060-0.125 μg/ml) (Table 2). In *S. prolificans *isolate 2, however, 40-65% of protoplasts were apoptotic after incubation with PCZ or ICZ in combination with TCR, and 35-70% were apoptotic after incubation with PCZ or ICZ with 17AAG (Table S1). We observed no annexin V-FITC staining in untreated protoplasts (Table 2). These results suggested that a fungicidal property of PCZ and ICZ was due to induction of apoptosis in *S. prolificans* cells, especially in combination with TCR or 17AAG.

**Table 2:** Percentage of *S. prolificans *(isolate 1) cells stained with annexin V, TUNEL and PI for detection of phosphatidylseriene exposure, DNA fragmentation and cell membrane integrity respectively. -, Not detected (0% of cells showed particular apoptotic marker) *MFC is given in parenthesis.

**Drugs (μg/ml)**	**Apoptotic protoplast %**		
	**Annexin V**	**TUNEL**	**PI**
Control	-	-	4.0±1.0
PCZ (64.0)	-	-	-
ICZ (64.0)	-	-	5.0±1.0
TCR (64.0)	-	-	3.0±1.0
17AAG (16.0)	-	-	3.0±1.0
PCZ + TCR (0.06 μg/ml)			
0.125	10.0±0.0	18.0±2.0	-
0.25	50.0±3.0	35.0±1.0	-
0.5	35.0±2.0	-	15.0±1.0
ICZ + TCR (0.125 μg/ml)			
0.060	23.0±2.0	12.0±1.0	3.0±1.0
0.125	30.0±1.0	35.0±1.0	5.0±1.0
0.25	40.0±2.0	40.0±2.0	15.0±1.0
PCZ + 17AAG (0.06 μg/ml)			
0.060	10.0±1.0	12.0±1.0	-
0.125	35.0±3.0	60.0±4.0	3.0±1.0
0.25	40.0±2.0	22.0±1.0	12.0±2.0
ICZ + 17AAG (0.125 μg/ml)			
0.060	5.0±1.0	20.0±1.0	-
0.125	30.0±1.0	50.0±4.0	5.0±0.0
0.25	30.0±2.0	10.0±1.0	15.0±0.0

To confirm the apoptotic features of PCZ and ICZ in *S. prolificans *germlings, we evaluated nuclear DNA fragmentation using a terminal deoxynucelotidyl transferase dUTP nick end labeling (TUNEL) assay. *S. prolificans *germlings exposed to PCZ or ICZ for 3 h at 37°C exhibited marked nuclear DNA fragmentation in a concentration-dependent manner (Table 2). The proportion of TUNEL-positive germlings was higher in both isolate 1 (50-60%) and isolate 2 (30-60%) in the presence of PCZ or ICZ (0.125 μg/ml) combined with 17AAG than when combined with TCR (isolate 1, 20-40%; isolate 2, 35-55%) (Tables 2 and S1).

### Induction of caspase-like activity in *S. prolificans* (isolate 1) germlings treated with PCZ or ICZ in combination with TCR or 17AAG

**Figure 4:**
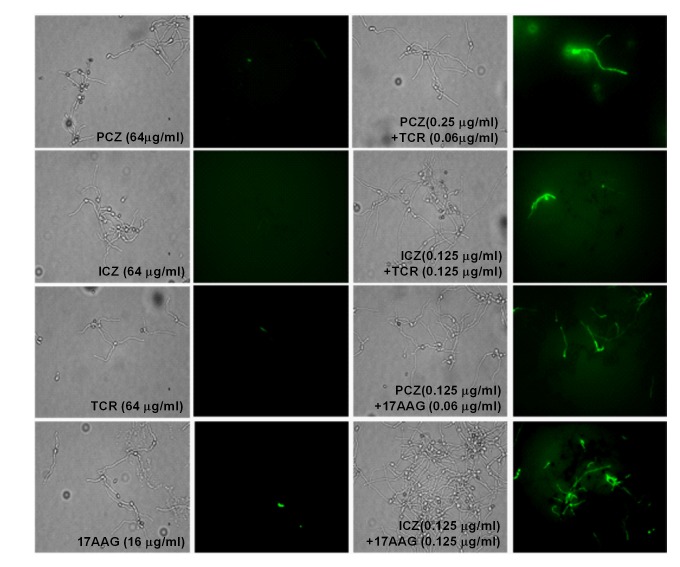
FIGURE 4: Detection of metacaspase (caspase-like) activity using CaspACE FITC-VAD-FMK probe in germlings of *S. prolificans *(isolate 1) treated with PCZ or ICZ in combination with TCR or 17AAG. Shown are fluorescent images of activated metacaspases of *S. prolificans *germlings treated with drugs alone and in combination with TCR or 17AAG.

Caspases are activated in the early stages of apoptosis and play a central role in the apoptotic cascade 23,24. Although caspases are not present in fungi, researchers have identified orthologs of mammalian caspases, called metacaspases in fungi 25. We stained *S. prolificans* germlings (isolate 1) pretreated with PCZ or ICZ in combination with TCR or 17AAG with the cell-permeable, broad-spectrum caspase inhibitor CaspACE-Z-VAD-FMK. In this staining, a green fluorescent signal is a direct measure of the amount of active caspase in a cell. *S. prolificans* germlings with activated metacaspases, treated with azoles in combination with TCR or 17 AAG were stained green, whereas germlings exposed to azoles alone remained unstained. This result indicated that treatment with PCZ or ICZ plus TCR or 17AAG triggered an apoptotic pathway in *S. prolificans* germlings via activation of metacaspases (Figure 4).

## DISCUSSION

We hypothesized that TCR and 17AAG enhance the negligible activity of the ergosterol biosynthesis inhibitors PCZ and ICZ, to the point that they become fungicidal, and that this fungicidal activity is mediated through apoptosis in *S. prolificans*. The calcineurin pathway and Hsp90 are important for the survival of pathogenic fungi because they have central roles in various cellular processes, including morphogenetic transition and development of antifungal tolerance and resistance 7,16. Inhibition of the calcineurin pathway and Hsp90 in combination with administration of conventional antifungal agents may have broad therapeutic potential in patients with fungal infections 16,26. Owing to the immunosuppressive properties of calcineurin inhibitors and the role of Hsp90 in controlling the calcineurin pathway, clinical use of a combination of TCR or 17AAG with triazole for treatment of *S. prolificans* infection would ultimately require a novel antifungal agent that selectively targets fungal stress pathways without having collateral effects on human immune cells.

We found evidence of synergy of PCZ and ICZ with TCR and 17AAG in *S. prolificans in vitro*, which is consistent with data on other fungal species 12,16,18,21. In addition, we used multiple markers of cell death to show that apoptosis is a mechanism of PCZ/ICZ- and TCR/17AAG-induced cell death. We corroborated the rate of apoptosis in *S. prolificans* germlings using assays for detection of phosphatidylserine (PS) by annexin V-FITC, ROS accumulation by DHR-123 staining and decreased mitochondrial membrane potential by Rh123, DNA damage by TUNEL staining, and activation of caspase-like activity by CaspACE FITC-VAD-FMK. In each of the assays, apoptosis was evident at PCZ, ICZ, TCR, and 17AAG concentrations (0.125-0.250 μg/ml) that were below the MIC of triazoles. Taken together, these data indicate that PCZ or ICZ combined with TCR or 17AAG at concentrations below the MIC causes apoptosis in *S. prolificans *germlings.

We found that induction of apoptosis and the fungicidal activity of PCZ and ICZ in combination with TCR or 17AAG correlated with increased plasma and mitochondrial membrane disruption, PS externalization, DNA fragmentation, and ROS accumulation in *S. prolificans* germlings (isolates 1 and 2) (Tables 1, 2 and S1, Figures 1-4). Calcineurin activity is known to contribute to the fungicidal effects of Hsp90 inhibitors. 17-AAG in particular induces apoptosis in colon carcinoma-derived cell lines 27, so determination of whether inhibition of Hsp90 can induce apoptotic cell death in fungi would be of interest. Dai *et al*. 28 demonstrated the role of Hsp90 in apoptosis in *C. albicans* and showed that inhibition of Hsp90 attenuated apoptosis by regulating the calcineurin pathway. Several fungi undergo apoptosis in response to antifungal treatment and various other stimuli 19. Additional studies providing better understanding of fungal apoptotic pathways would promote the discovery of much-needed antifungal therapies.

Our results indicated that disruption of mitochondrial integrity by a combination of PCZ/ICZ with TCR or 17AAG induced apoptosis in *S. prolificans*. Our study in *S. prolificans *and studies in other fungi showed that 17AAG inhibits Hsp90, causing mitochondria-mediated apoptosis in rat histiocytomas 29. Also, Shirazi and Kontoyiannis 18 showed that increased apoptosis after exposure to TCR was correlated with increased intracellular ROS accumulation in Mucorales. Furthermore, translocation of mitochondrial cyt *c* to the cytosol has led to binding of cyt *c* with apoptotic protease-activating factor to form a complex with caspase-9, resulting in caspase activation 23,24,30. Release of cyt *c* requires an increase in mitochondrial membrane permeability during apoptosis 23. As in Mucorales, our results in *S. prolificans* also demonstrated that, ROS formation, changes in ΔΨ_m_, and cyt *c* release were associated with apoptosis 20,21,22. Authors have also reported ROS-induced apoptosis in *A. nidulans*, *Fusarium oxysporum*, and *C. albicans* 31,32,33. Sharon *et al*. 25 reported that apoptotic pathways in fungi seem to be mitochondrion-dependent, and can be powerful sources of superoxide radicals in cells undergoing miconazole and farnesol-induced apoptosis 34.

Authors have reported accumulating evidence that different stimuli induce different apoptotic pathways in yeasts and other fungi 35,36. In mammals, apoptosis is regulated by activation of caspases, which cleave specific substrates and trigger apoptotic death 37. Now it is evident that caspase-like proteolytic activity may exist not only in multicellular organisms but also unicellular organisms, such as fungi. In the current study, we observed caspase-like activity in *S. prolificans* germlings upon exposure to PCZ or ICZ with TCR or 17AAG. Further studies are needed to demonstrate how proteases contribute to apoptotic fungal death.

In conclusion, we have shown for the first time that co-administration of inhibitors of the ergosterol biosynthesis pathways with an inhibitor of calcineurin or Hsp90 induces apoptosis in the recalcitrant fungus *S. prolificans*. This fungicidal synergistic interaction requires further study, as it may be a useful therapeutic strategy for infections caused by pathogenic fungi for which treatment options are extremely limited.

## MATERIALS AND METHODS

### Drugs

PCZ stock (5 mg/ml; Merck & Co., Inc.) was prepared in distilled water. ICZ (5 mg/ml; Janssen Pharmaceuticals), TCR (1 mg/ml; Medisca), and 17AAG (Sigma) stocks were prepared in ethanol, and aliquots were stored at -20°C in the dark until use.

### Isolates and growth conditions 

Three clinical isolates of *S. prolificans *(*S.p*-071507 [isolate 1], 071826 [isolate 2], and 674802 [isolate 3]) were grown on freshly prepared Sabouraud dextrose agar plates. After 48 h of incubation at 37°C, spores were collected and washed twice in sterile phosphate-buffered saline (PBS). The spores were then counted using a hemocytometer and stored at 4°C in PBS.

### Susceptibility testing

Broth microdilution was performed according to the Clinical and Laboratory Standards Institute method 38. Briefly, two-fold serial PCZ and ICZ dilutions were prepared in flat-bottomed 96-well microtiter plates (100 μl/well) in the presence or absence of TCR or 17AAG (0.060-0.125 µg/ml). Drug-free wells were used as controls. Each well was inoculated with 100 μl of freshly isolated *S. prolificans* spores (2-3 days old; 1 × 10^4^ spores/ml) suspended in RPMI medium. After 48 h of incubation at 37°C, the MICs of PCZ and ICZ were determined visually as the lowest drug concentrations resulting in complete growth inhibition. To determine the minimum fungicidal concentrations (MFC) of PCZ and ICZ, an aliquot (20 μl) from each well that exhibited 100% growth inhibition was plated onto YPD agar (1% yeast extract, 2% peptone, 2% dextrose and 2% agar) plates. After 24 h of incubation at 37°C, the MFC was recorded as the lowest drug concentration at which no growth was observed.

For all of the wells of the microtiter plates that corresponded to MICs, the sum of the fractional inhibitory concentrations (ΣFIC) was calculated for each well using the equation ΣFIC = FICA + FICB = (CA/MICA) + (CB/MICB), in which MICA and MICB are the MICs of drugs A and B alone, respectively, and CA and CB are the concentrations of the drugs in combination, respectively, in all of the wells corresponding to an MIC. Synergy was defined as a ΣFIC of up to 0.5. Indifference was defined as a ΣFIC of at least 0.5 but no more than 4.0. Antagonism was defined as a ΣFIC greater than 4.0.

### Viability assay 

*S. prolificans* germlings (isolates 1 and 2) treated with TCR or 17AAG (0.060-0.125 μg/ml) along with PCZ (0.06-0.50 μg/ml) or ICZ (0.06-0.25 μg/ml) for 3 h were stained with DiBAC (Molecular Probes) as described previously 21,22.

### Annexin V-FITC-PI double staining of *S. prolificans *(isolates 1 and 2)

The apoptosis marker PS is located on the inner leaflet of the lipid bilayer of the cytoplasmic membrane and is translocated to the outer leaflet at the onset of apoptosis 39,40,41. PS can be detected using staining with annexin V-FITC, which binds to it. Germlings treated with PCZ (0.06-0.50 μg/ml) or ICZ (0.06-0.25 μg/ml) in combination with TCR or 17AAG (0.060 and 0.125 μg/ml) were digested with a lysing enzyme mixture (0.25 mg/ml chitinase, 15 U of lyticase, and 20 mg/ml lysing enzyme; Sigma) for 3 h at 30°C. After digestion, *S. prolificans *protoplasts were stained with annexin V-FITC (BD Pharmingen) and PI at room temperature for 15 min and observed under a fluorescence microscope to assess the externalization of PS as described previously 39.

### Detection of intracellular ROS accumulation and ΔΨ_m_ in *S. prolificans* germlings (isolates 1 and 2)

ROS plays an important role as an early initiator of apoptosis in yeasts and other filamentous fungi 20,21,22. The amount of ROS in *S. prolificans* germlings was measured using DHR-123 (Sigma) staining 20,21,22. The mitochondrial membrane potential was assessed by staining with Rh-123 (Sigma), a fluorescent dye that diffuses in the matrix in response to electric potential as described 20,21,22. Intracellular ROS levels and ΔΨ_m_ in *S. prolificans* germlings were measured after treatment with PCZ (0.060-0.50 μg/ml) or ICZ (0.060-0.25 μg/ml) in combination with TCR and 17 AAG (0.060 and 0.125 μg/ml) for 3 h at 37°C using a fluorimetric assay with DHR-123 and Rh-123 staining 20,21,22,42.

### Measurement of DNA damage in *S. prolificans* (isolates 1 and 2)

DNA fragmentation, a characteristic of apoptosis, was detected in *S. prolificans* using a TUNEL assay. Germlings pretreated with PCZ (0.06-0.50 μg/ml) or ICZ (0.06-0.25 μg/ml) in combination with TCR or 17AAG (0.060 and 0.125 μg/ml) for 3 h at 37°C were fixed with 3.7% formaldehyde for 30 min on ice and digested using a lysing enzyme mixture. Enzyme-digested germlings were used to detect DNA fragmentation using a TUNEL assay as described by Madeo *et al.* 41. The protoplasts were observed for fluorescence with excitation and emission wavelengths of 488 nm and 520 nm, respectively.

### Detection of metacaspase activity using CaspACE FITC-VAD-FMK in *S. prolificans* germlings (isolates 1 and 2)

Active metacaspases in *S. prolificans* germlings were detected using CaspACE FITC-VAD-FMK (Promega) according to the manufacturer's instructions 20,21,22. Briefly, germlings pretreated with PCZ (0.06-0.50 μg/ml) or ICZ (0.06-0.25 μg/ml) in combination with TCR or 17AAG (0.060 and 0.125 μg/ml) for 3 h at 37°C were collected, washed in PBS, resuspended in 10 μM FITC-VAD-FMK, and incubated again for 2 h at 30°C. Apoptosis in the *S. prolificans* germlings was inhibited in the presence of the caspase inhibitor z-VAD-FMK (Sigma) at final concentrations of 40 μM. After incubation, germlings were washed twice in PBS and observed microscopically for fluorescence with excitation and emission settings of 488 nm and 520 nm, respectively.

### Statistical Analysis

For all assays, three independent experiments were performed in triplicate. Comparisons of multiple treatment groups were performed by using two-way analysis of variance with post-hoc paired comparisons using Dunnett’s test. Calculations were made using the InStat software program (GraphPad Software). Two-tailed *P* values of less than 0.05 were considered statistically significant.

## SUPPLEMENTAL MATERIAL

Click here for supplemental data file.

All supplemental data for this article are also available online at http://microbialcell.com/researcharticles/heat-shock-protein-90-and-calcineurin-pathway-inhibitors-enhance-the-efficacy-of-triazoles-against-scedosporium-prolificans-via-induction-of-apoptosis/.
