# Supplementary material for: Heat shock protein 90 and calcineurin pathway inhibitors enhance the efficacy of triazoles against Scedosporium prolificans via induction of apoptosis
Source: Microb Cell. 2014 Jun 2;1(6):179–88. doi: 10.15698/mic2014.06.150 (PMC5354560; doi:10.15698/mic2014.06.150)
Supplement: Supplementary file 1 [file mic-01-179-s01.pdf]

Table S1. Percentage of apoptotic cells and percent fold change in fluorescence in *S. prolificans* germlings (isolate-2) stained with annexin V, TUNEL, PI, DiBAC, DHR-123 and Rh-123.

**Drugs (µg/ml)**	**Apoptotic cells**					
	**Apoptotic protoplast %**			**% Fold change**		
	**Annexin V**	**TUNEL**	**PI**	**DiBAC**	**ROS**	**Ψm**
PCZ + TCR (0.06 µg/ml)						
0.125	50.0±2.0	40.0±3.0	2.0±0.0	1.1±0.0	1.0±0.0	1.3±0.1
0.25	60.0±4.0	40.0±2.0	-	1.4±0.1	1.3±0.0	1.8±0.0
0.5	40.0±1.0	15.0±1.0	20.0±1.5	2.0±0.0	1.2±0.1	1.4±0.0
ICZ + TCR (0.125 µg/ml)						
0.060	65.0±5.0	35.0±4.0	10.0±1.0	1.2±0.0	1.4±0.0	1.0±0.0
0.125	65.0±5.0	55.0±5.0	10.0±1.0	1.3±0.2	1.3±0.0	1.4±0.2
0.25	40.0±2.0	45.0±4.0	25.0±2.0	1.9±09.0	1.0±0.0	1.3±0.0
PCZ + 17AAG (0.06 µg/ml)						
0.060	70.0±1.0	60.0±2.0	5.0±1.0	1.1±0.0	1.4±0.2	1.0±0.0
0.125	50.0±2.0	55.0±5.0	35.0±1.5	1.5±0.3	2.1±0.0	1.7±0.0
0.25	50.0±5.0	30.0±3.0	10.0±1.0	1.4±0.2	1.8±0.0	2.1±0.5
ICZ + 17AAG (0.125 µg/ml)						
0.060	65.0±5.0	50.0±1.0	5.0±0.0	1.1.±0.0	1.0±0.0	1.0±0.0
0.125	40.0±4.0	35.0±1.0	15.0±1.0	1.4±0.0	1.9±0.0	1.2±0.0
0.25	35.0±3.0	20.0±2.0	15.0±1.0	2.0±0.0	1.7±0.0	1.2±0.0

-, Not detected (0% of cells showed particular apoptotic marker); TCR or 17AAG control showed ≤1 fold increase in fluorescence in comparison with triazoles alone.
